# Human papillomavirus prevalence in invasive cervical carcinoma by HIV Status

**DOI:** 10.1186/1750-9378-7-S1-O12

**Published:** 2012-04-19

**Authors:** Hugo De Vuyst, Gathari Ndirangu, Manivasan Moodley, Vanessa Tenet, Benson Estambale, Chris JLM Meijer, Peter JF Snijders, Gary Clifford, Silvia Franceschi

**Affiliations:** 1 International Agency for Research on Cancer, Lyon, France; 2 Kenyatta National Hospital, University of Nairobi, Nairobi, Kenya; 3 University of KwaZulu-Natal, Durban, South Africa; 4 University of Nairobi, Institute of Tropical and Infectious Diseases, Nairobi, Kenya; 5 Department of Pathology, VU University Medical Center, Amsterdam, The Netherlands

## Background

Data on the prevalence of human papillomavirus (HPV) types in invasive cervical carcinoma (ICC) in women with HIV are scarce but are essential to elucidate the influence of immunity on the carcinogenicity of different HPV types, and the potential impact of prophylactic HPV vaccines in populations with high HIV prevalence.

## Objectives

To compare the prevalence of HPV types in ICC by HIV status.

## Methods

From 2007 to 2009, a multicentre case-case study was conducted at two referral hospitals in Nairobi, Kenya, and in Durban, South Africa. Women over 18 years of age presenting with ICC were recruited, and frozen biopsies were obtained and tested for HPV DNA using GP5+/6+-PCR methodology. The present analysis was limited to the 235 squamous cell cancers (SCC) detected.

## Results

**Figure 1:**
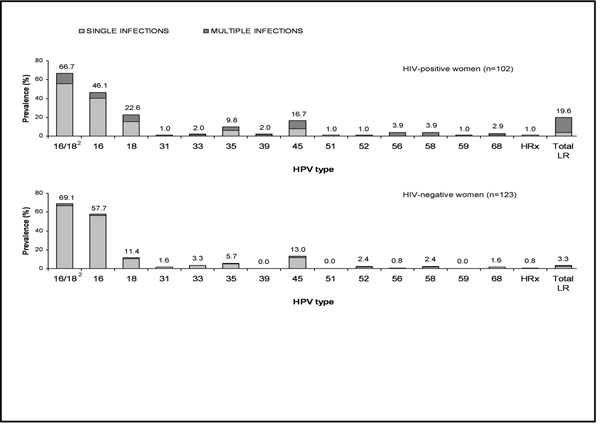
Prevalence of human papillomavirus (HPV) in 225 women with cervical squamous cell carcinoma by HIV status and multiplicity of HPV infection^^1^10 HPV-negative women were excluded; ^2^Either 16 or 18 as single infection or in combination with any type as multiple type infection; HPV: human papillomavirus; HRX: uncharacterized high-risk type; LR: low-risk.^

We included 106 HIV-positive (mean age 40.8 years) and 129 HIV-negative women (mean age 45.7) with SCC. Among HIV-positive women, the mean CD4 count was 334 cells/μL and 48.1% were on combined antiretroviral therapy. HIV-positive women had many more multiple HPV infections (21.6% of HPV-positive carcinomas) compared to HIV-negative women (3.3%) (p <0.001) and the proportion of multiple infections was inversely related to CD4 level. An excess of HPV18 of borderline statistical significance was found in HIV-positive compared to HIV-negative women (Prevalence ratio (PR) = 1.9, 95% confidence interval (CI): 1.0-3.7, adjusted for centre, age and multiplicity of infection). HPV16 and/or 18 prevalence combined, however, was similar in HIV-positive (66.7%) and HIV-negative women (69.1%) (PR = 1.0, 95% CI: 0.9-1.2). No significant difference was found for other HPV types (Figure 1).

## Conclusions

Overall, our data suggest that current prophylactic HPV vaccines against HPV16 and 18 may prevent similar proportions of cervical SCC in HIV-positive as in HIV-negative women provided that vaccine-related protection is sustained after HIV infection.

